# A new micro swim-up procedure for sperm preparation in ICSI treatments: preliminary microbiological testing

**DOI:** 10.5935/1518-0557.20160023

**Published:** 2016

**Authors:** Simone Palini, Mariangela Primiterra, Silvia De Stefani, Maria Federica Pedna, Monica Sparacino, Patrizia Farabegoli, Serena Benedetti, Carlo Bulletti, Vittorio Sambri

**Affiliations:** 1 Cervesi Hospital, IVF Unit, AUSL Romagna, Italy; 2 Unit of Microbiology, The Greater Romagna Hub Laboratory, AUSL della Romagna, Pievesestina, Cesena, Italy; 3 University of Urbino "Carlo Bo", Department of Biomolecular Sciences, Section of Clinical Biochemistry and Molecular Genetics, Urbino, Italy; 4 DIMES, University of Bologna, Bologna, Italy

**Keywords:** ICSI, micro swim-up method, microbial contamination, semen treatment

## Abstract

**Objective:**

This study aimed to assess the levels of microbial contamination in semen samples before and after the micro swim-up (MSU) procedure in intra-cytoplasmic sperm injection (ICSI). The new method is an upgrade to the classic wash swim-up procedure.

**Methods:**

Semen analysis and microbiological tests were carried out before and after the MSU procedure. A total of twenty semen samples were analyzed.

**Results:**

Pathogens were observed in semen samples only before MSU and never after ICSI. Microbiological tests revealed a large prevalence of gram-positive cocci [Staphylococcus spp. (n=16, 80%) and viridans streptococci (n=10, 50%)]. The results of this study indicate that direct MSU in ICSI improved the ICSI workflow.

**Conclusion:**

The new workflow is faster and more affordable, and is likely to prevent infection problems that could arise from the normal microbial flora of the semen.

## INTRODUCTION

Human ejaculate is a mixture of seminal plasma, mature and immature spermatozoa, non-reproductive cells, non-specific debris and various microorganisms. As a consequence, semen is not sterile and may contain microorganisms even after washing (Al-Mously *et al*., 2009; Campos *et al.,* 2012; Cottell *et al.,* 2000; Knox *et al*., 2003; Michou *et al.,* 2012). Semen bacterial infections may significantly jeopardize one's fertility status and reproductive potential (Fraczek *et al*., 2014; Moretti *et al*., 2009). Accordingly, ejaculated samples from patients with genital tract infections present deteriorated semen volume, sperm concentration, motility, morphology, and vitality (Sanocka-Maciejewska *et al.,* 2005; Weidner *et al*., 2013).

Even when most of the microorganisms detected in the semen samples are nonpathogenic commensals or contaminants, their presence may be of greater significance in in vitro fertilization (IVF), a scenario in which the natural defenses of the female genital tract are bypassed (Cottell *et al.,* 2000; Hewitt *et al*., 1985). Contamination of the culture system with seminal microbes may lead to suboptimal fertilization rates or impaired embryonic development (Guillet-Rosso *et al.,* 1987; Shalika *et al.,* 1996) when adequate seminal processing techniques with an antibiotic-rich culture medium are not used (Bielanski, 2007; Cottell *et al*., 1997).

Several selection methods have been recently developed to improve sperm preparation in IVF protocols to allow the segregation of mature, structurally intact, and non-apoptotic spermatozoa with high DNA integrity (Said & Land, 2011). Among these, gradient centrifugation followed by a swim-up procedure is known to effectively reduce contamination; however, the number and species of identified microorganisms may vary greatly (Adiga & Kumar, 2001) and possible adverse effects such as the development of reactive oxygen species (ROS) during centrifugation may occur (Shekarriz *et al.,* 1995), contributing to spermatozoa DNA damage (Aitken & Clarkson, 1988).

Different approaches have been proposed to reduce the microbial contamination of the culture medium by seminal fluid. The first involves the counseling of the partner about a sterile technique for semen collection. Nicholson *et al.* (2000) found that if pipettes and tubes were changed in every stage of the procedure, the contamination could be eliminated. In addition, Kastrop *et al*. (2007) reported that infections were observed only in IVF culture dishes and never after intra-cytoplasmic sperm injection (ICSI), thus indicating that the ICSI procedure prevented culture dish colonization by microorganisms.

Over the past few years, ICSI has become a more popular procedure than conventional IVF in Europe (de Mouzon *et al*., 2010) and the most important option in human assisted reproduction technology (ART). Polyvinylpyrrolidone (PVP) can be used to regulate the viscosity in the injection pipette and to limit the final volume injected into the oocyte (Gianaroli *et al.,* 2000); however, adverse effects following the use of this artificial polymer have been reported (Barak *et al*., 2001). The level of PVP purification and the potential for contamination may be critical in the generation of more efficient techniques for human ICSI (Kato & Nagao, 2012).

The present study investigated a modification of the classic wash swim-up procedure. The aim was to assess the level of microbial contamination in semen samples before and after the micro swim-up (MSU) procedure in ICSI, and to collect evidence concerning the possible differences between patients submitted or not to water loading before sperm collection, as reported in the World Health Organization guidelines (WHO, 2010). The ultimate purpose was to verify whether direct MSU was a valid procedure in ICSI.

## MATERIALS AND METHODS

### Study population

The study included twenty patients (age 38.9±5.4 years, range 28-49 years) admitted into the Unit of Pathophysiology of Reproduction (Cervesi Hospital, Cattolica, RN, Italy) for semen analysis. The enrolled individuals answered a questionnaire and provided data on their histories of urinary tract infection, genital tract surgery, and drug treatment; the group offered water loading also had to report the number of urinations before ejaculation. Individuals diagnosed with azoospermia or given antibiotics up to four weeks before the start of the study were excluded. Only semen samples with a concentration > one million sperm/ml without considering other seminal features were included, since the principal outcome was to investigate the microbial contamination of the samples. The institution's Internal Review Board approved the study (permit 09/2014). The samples used were leftovers from normal practice. The patients consented to having their residual samples used for research purposes. The samples were anonymized before testing.

### Semen collection and microbiological investigation

Semen samples were collected in sterile plastic containers by masturbation without using condoms or lubricants after three days of abstinence as recommended by WHO guidelines (2010). The patients were randomly divided into two groups: one group received a water load before the collection of semen, while the other one was left on a free water intake regimen. The patients were asked to wash their hands and genital areas with soap and water, and then to dry both hands and genital areas before sperm collection.

The semen donors selected for the study were checked for previous and active infections by HIV, HCV, HBV, and Treponema pallidum by standard serological techniques and RT-PCR (Abbott Molecular, Abbott Park, Ill., USA). Additionally, a specific evaluation was carried out to investigate the presence of specific sexually transmitted pathogens in the semen specimens. A multiplex real time PCR technique (Seegene, Seoul, R. of Korea) capable to simultaneously identify Ureaplasma urealyticum, Mycoplasma hominis, Mycoplasma genitalium, Ureaplasma parvum, Neisseria gonorrhoeae, Chlamydia trachomatis, and Trichomonas vaginalis was used. A standard bacterial culture (based on inoculation of blood and "chocolate" agar plates and Brain-Heart Infusion broth, followed by incubation at 37°C for up to 72 hours) was also performed in order to investigate the presence and amount of colonizing bacteria delivered with the semen at ejaculation.

### Semen analysis

After liquefaction at 37°C (10-minute minimum, 30-minute maximum) and under sterile conditions, the semen samples were mixed with a sterile pipette and analyses were performed according to the guidelines of the WHO (2010). After routine semen clinical tests, an aliquot was removed for microbiological and PCR semen analysis.

### Sperm selection procedure

**Figure 1:**
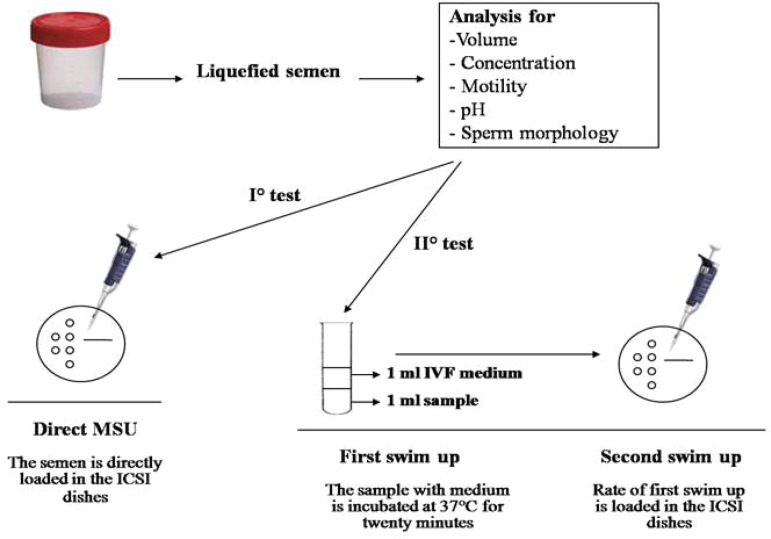
Sperm swim-up procedures.

For sperm selection, two different swim-up procedures were tested: I) a direct MSU and II) a first swim-up in G-IVF^TM^ PLUS medium followed by MSU (Figure 1). In the first test, the ejaculate was directly placed on the ICSI dish without additional treatments. In the second test, the semen sample (1 mL) was incubated for twenty minutes at 37°C under 6% CO2 in G-IVF^TM^ PLUS (1 mL) for the first swim-up, and then submitted to the second migration by MSU.

**Figure 2:**
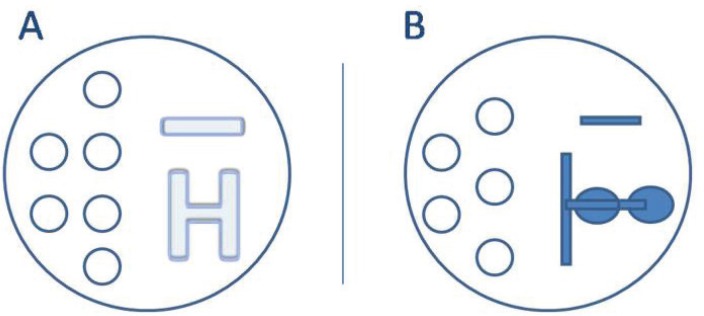
Spermatozoa migration patterns. A) H pattern; B) Two serial drop pattern.

Two different patterns of spermatozoa migration were used in the tests mentioned above. The dish was prepared with droplets of handling medium for oocytes (droplet volume 10 µL), and an H pattern (Figure 2A) or a two serial drop pattern (Figure 2B) were produced, followed by an ICSI^TM^ (Vitrolife) for semen. Finally, all the dishes were covered with OVOIL^TM^ (Vitrolife). ICSI^TM^ is a viscous sperm handling solution containing PVP and recombinant human albumin. Isolating single motile spermatozoa from the PVP solution during the ICSI procedure may reduce the risk of contamination.

Two different set of ICSI were prepared: one ICSI microplate using the real volumes needed for the ICSI procedure, and one ICSI macroplate with greater volumes.

### ICSI Microplate

**Figure 3:**
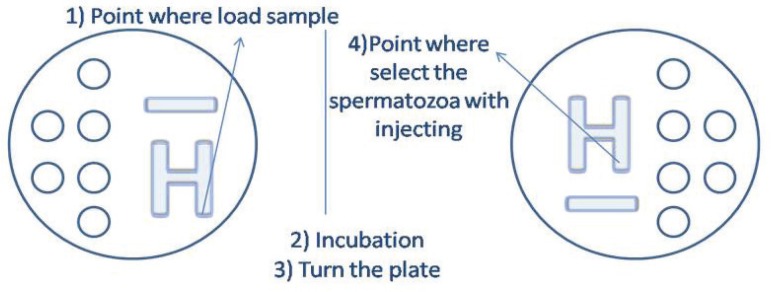
ICSI microplate with H migration pattern.

The ICSI^TM^ was used to produce the H pattern (length of about 2 cm from one side of the plate to the middle of the disk, total volume 10 µL). A small amount of semen (3 µL) was placed on one side of the H, and some time was allowed for it to migrate to the other side where the sperm was then collected. The dishes were incubated for three minutes allowing sperm migration along the outer perimeter of the H. After the completion of sperm migration, the plate was turned before the upload to avoid the contamination of the injection pipette by the sample (Figure 3). The injection pipette was introduced into the H-ICSI^TM^ and used to immobilize a single sperm by crushing the membrane of its tail. The sperm was collected and transported to a new drop of ICSI^TM^ buffer simulating the ICSI technique and releasing the same amount of volume used in a hypothetical ICSI. The ICSI^TM^ medium and the Gamete buffer used for ICSI injection, microbiological and PCR testing in both samples were then analyzed.

**Figure 4:**
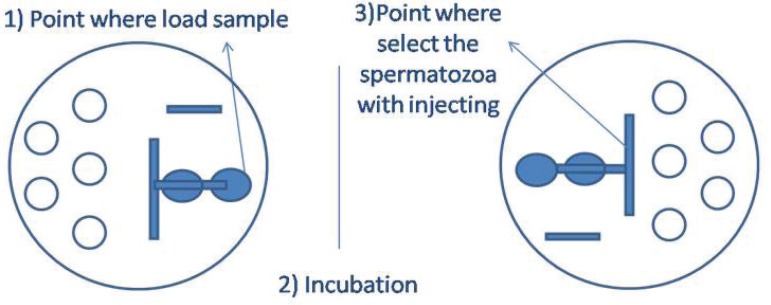
ICSI microplate with two serial drop migration pattern.

The same volumes were applied in the second migration pattern. In this case, the long path allowed at first semen dilution and subsequently PVP migration. As described above, the semen samples were placed and incubated in ICSI dishes; and before sperm selection, the dishes were turned (Figure 4).

### ICSI Macroplate

Macro experiments were also performed to confirm the ICSI microplate results. Sperm samples with increasing volumes (37.5/75/150µL) were placed onto 125/250/500µL of ICSI^TM^, respectively, following the H pattern or the two serial drop pattern. Greater volumes for microbiological investigation by PCR analysis were thus obtained. The dishes were then incubated for a period of ten to twelve minutes. Sperm selection was performed using the same procedure described for the ICSI microplate. The ICSI^TM^ present on the edge of the H or of the drop was collected and submitted to microbiological analysis. Aliquots for PCR testing were stored at -20°C.

### Statistical analysis

The differences in semen bacterial contamination between patients submitted and individuals not submitted to water loading were assessed by the Chi-square test. Statistical significance was attributed to differences with a *P*< 0.05.

## RESULTS

### Microbiological testing before semen selection procedures

After collection, the semen samples were tested for the presence of bacterial contamination. The results were summarized in Table 1. All the samples showed variable levels of contamination, with a large prevalence of a mixed bacterial flora, as expected. The vast majority presented multiple contaminations: Staphylococcus spp. were the most frequent contaminants, being present in 80% (16/20) of the samples, followed by viridans streptococci (10/20, 50%). The presence of gram-negative rods was less relevant (overall 5/20, 25%), as was the number of specimens showing the presence of Enterococcus spp (2/20, 10%).

**Table 1:** Incidence of semen bacterial contamination in patients submitted or not to water loading before semen collection.

Bacteria	Total (n=20)	Water loading (n=10)	No water loading (n=10)	*P* values
Staphylococcus spp.	16	9	7	0.264
Viridans streptococci	10	5	5	1
Gram-negative bacilli (not identified)	3	1	2	0.531
Proteus mirabilis	1	1	0	0.305
Escherichia coli	1	0	1	0.305
Enterococci	2	1	1	1
**TOTAL**	**33**	**17**	**16**	**0.768**

Overall, the incidence of bacterial contamination was similar in patients submitted to water loading versus individuals not offered water loading (17 vs. 16 total cases of contaminating bacteria, *P*=0.768). No significant differences were observed in the distribution of species of bacteria between patients given water loading and individuals not offered water loading.

PCR analysis for HIV, HBV, and HCV showed absence of viruses in semen samples. One semen sample was found to be positive for the presence of Ureaplasma parvum, whereas another showed the contemporary presence of Ureaplasma parvum and Chlamydia tracomatis.

### Microbiological testing after semen selection procedures

The same microbiological analysis was repeated after the different procedures of semen selection. The cultures and PCR analyses of the samples submitted to either of the selection procedures (i.e. direct MSU vs. swim-up in G-IVF^TM^ PLUS medium followed by MSU) or spermatozoa migration patterns (H vs. two serial drops) showed no microbiological contamination.

## DISCUSSION

Efforts have been made to develop physiological and minimally invasive human IVF procedures. The literature has indicated that repeated centrifugation in semen washing partially removes pathogens inherent to the semen (Knox *et al*., 2003; Michou *et al.,* 2012; Shalika *et al*., 1996) and increases sperm DNA fragmentation (Robinson *et al*., 2012). Moreover, Hadnott *et al.* (2015) reported cases of embryo aneuploidy derived from both IVF and ICSI procedures pushing to improve or modify the preparation of gametes.

The present study looked into a modification of the classic wash swim-up procedure and assessed the microbial contamination in semen samples before and after the micro swim-up (MSU) procedure in ICSI. First of all, our study found no significant differences in the incidence of semen bacterial contamination or in the distribution of bacterial species between patients submitted or not to water loading before semen collection. Secondly, the two MSU techniques yielded positive results in terms of absence of contamination. Incidentally, the first swim-up followed by a second swim-up in PVP resulted in more safety due to the dilution of the semen sample and the presence of antibiotics in the dilution medium to further decrease the presence of residual microbial infection. Interestingly, both patterns of spermatozoa migration - the H and the two serial drop pattern - may be used in ICSI, since no contamination was found in the ICSI dish.

The proposed MSU procedure is safe and does not allow sperm capacitation. However, our preliminary data show that sperm activation through immobilization before ICSI may be enough to enable the fertilization process. Indeed, damage to the sperm plasma membrane has been described as a necessary process prior to ICSI, as it plays a key role in oocyte activation caused by spermatozoa (Dozortsev *et al.*, 1995; Palermo *et al*., 1996).

In conclusion, our study found that in the MSU procedure semen samples can be used directly for ICSI without the risk of bacterial contamination. This technique reduces the time of manipulation as well as the cost of semen treatment and might still allow dramatic decreases in the incidence of possible mismatches during tube preparation. This study opens the door to the introduction of MSU in IVF treatments. Subsequent studies are being designed and will be published to prove its efficacy in terms of fertilization, embryo development and pregnancy.

Abbreviations: ART: assisted reproduction technology; ICSI: intra-cytoplasmic sperm injection; IVF: in vitro fertilization; MSU: micro swim-up, PCR: polymerase chain reaction; PVP: polyvinylpyrrolidone, ROS: reactive oxygen species.
